# Sleep-Disordered Breathing and Behavioral Symptoms in Pediatric Orthodontic Patients: A Multicenter Cross-Sectional Study

**DOI:** 10.3390/jcm15093386

**Published:** 2026-04-29

**Authors:** Valeriu Mihai But, Sorana Nicoleta Roșu, Cristina-Ioana Bica, Alexandru Vlasa, Tatiana-Maria Coman, Clara Diana Haddad, Alexandra Mihaela Stoica, Mariana Pacurar, Mahmoud Elsaafin

**Affiliations:** 1 Department of Medicine-Psycho-Neuroscience and Recovery, Faculty of Medicine and Pharmacy, University of Oradea, 410073 Oradea, Romania; dr.butvaleriu@gmail.com; 2 Department of Oral and Maxillofacial Surgery, Faculty of Dentistry, Grigore T. Popa University of Medicine and Pharmacy, 700115 Iași, Romania; 3 Department of Pedodontics, George Emil Palade University of Medicine, Pharmacy, Science, and Technology of Târgu Mureș, Gh Marinescu 38, 540142 Târgu Mureș, Romania; cristina.bica@umfst.ro; 4 Department of Periodontology, Faculty of Dental Medicine, George Emil Palade University of Medicine, Pharmacy, Science, and Technology of Târgu Mureș, 38 Gh. Marinescu Str., 540142 Târgu Mureș, Romania; alexandru.vlasa@umfst.ro; 5 Department of Orthodontics, Faculty of Dental Medicine, George Emil Palade University of Medicine, Pharmacy, Science, and Technology of Târgu Mureș, 38 Gh. Marinescu Str., 540142 Târgu Mureș, Romania; tatiana-maria.coman@umfst.ro (T.-M.C.); mariana.pacurar@umfst.ro (M.P.); nabil.elsaafinmahmoud@umfst.ro (M.E.); 6 Department of Orthodontics, Faculty of Dentistry, Grigore T. Popa University of Medicine and Pharmacy, 700115 Iași, Romania; haddad.claradiana95@gmail.com; 7 Department of Odontology and Oral Pathology, George Emil Palade University of Medicine, Pharmacy, Science, and Technology of Târgu Mureș, 540139 Târgu Mureș, Romania; alexandra.stoica@umfst.ro

**Keywords:** sleep apnea, obstructive, mouth breathing, pediatric orthodontics, attention deficit disorder with hyperactivity, behavioral symptoms, pediatric sleep questionnaire, airway screening

## Abstract

**Background/Objectives**: Sleep-disordered breathing (SDB), including obstructive sleep apnea, is common in children and is associated with mouth breathing, snoring, and neurobehavioral disturbances. In pediatric orthodontic patients, oral habits and craniofacial imbalances may contribute to airway dysfunction, making orthodontic evaluation a potential setting for early identification of SDB. This study aimed to estimate the prevalence of SDB and to evaluate its associations with parent-reported behavioral symptom profiles in a cohort of pediatric orthodontic patients. **Methods**: A multicenter cross-sectional study was conducted in 186 children aged 7–13 years attending orthodontic clinics in Oradea and Târgu Mureș, Romania. Parents completed a structured questionnaire on oral habits, the 22-item Pediatric Sleep Questionnaire (PSQ), with SDB defined as 8 or more positive responses, and a parent-reported behavioral screening form assessing ADHD symptom subtypes, oppositional-defiant disorder (ODD), conduct disorder, and anxiety/depression. These behavioral outcomes were based on screening measures and were not intended as clinical psychiatric diagnoses. Associations were analyzed using chi-square or Fisher’s exact tests, and multivariable logistic regression analyses were performed adjusting for age, sex, and weight status. **Results**: Mouth breathing was reported in 61.8% of participants, snoring in 26.9%, and SDB in 13.4%. Positive screens for ADHD-inattentive (*p* < 0.001), ADHD-hyperactive/impulsive (*p* < 0.001), ADHD-combined (*p* < 0.001), ODD (*p* < 0.001), and anxiety/depression (*p* < 0.001) were significantly more frequent among children with SDB. In multivariable analysis, SDB remained independently associated with ADHD-combined subtype (OR = 6.22), ADHD-hyperactive/impulsive symptoms (OR = 5.84), oppositional-defiant disorder (OR = 4.91), and anxiety/depression (OR = 4.38). **Conclusions**: SDB was identified in a meaningful proportion of pediatric orthodontic patients and was significantly associated with multiple screening-defined behavioral symptom domains. These findings support consideration of brief airway- and sleep-oriented screening during orthodontic assessment, particularly in school-aged children presenting with mouth breathing, snoring, or behavioral concerns. Given the cross-sectional and questionnaire-based design, the findings should be interpreted as associative and warrant confirmation in prospective studies using objective sleep measures.

## 1. Introduction

Sleep-disordered breathing (SDB) includes a spectrum of nocturnal respiratory disturbances ranging from primary snoring to obstructive sleep apnea and affects an estimated 10–15% of children [1,2]. Its clinical relevance stems from its strong association with neurocognitive impairment, daytime irritability, reduced school performance, learning difficulties, and behavioral disturbances [3]. Among these, attention-deficit/hyperactivity disorder (ADHD)-like symptoms are frequently reported, leading to diagnostic confusion and, in some cases, unnecessary psychostimulant therapy. Early identification of SDB in pediatric populations is therefore crucial [1].

Orthodontic practice represents a key setting for recognizing early signs of airway dysfunction. Children attending orthodontic clinics frequently present craniofacial imbalances, malocclusion patterns, and parafunctional oral habits that may influence airway patency. Parafunctional oral habits are highly prevalent in children, with international studies reporting rates between 20% and 60% depending on age and habit type [4,5]. Oral habits such as mouth breathing, atypical swallowing, bruxism, lip incompetence, and mandibular postural habits can alter the balance between orofacial muscles, dentition, and skeletal structures [6]. When these behaviors persist beyond early childhood, they may contribute to maxillary constriction, increased lower anterior facial height, and abnormal occlusal development. Certain malocclusion patterns, such as maxillary constriction and retrognathia, have also been linked to reduced upper-airway dimensions and increased susceptibility to SDB [7]. Maxillary constriction may reduce the transverse dimensions of the nasal floor and increase nasal resistance, thereby contributing to impaired upper-airway patency.

Among all habits, chronic mouth breathing is particularly important because it directly affects nasal airflow, modifies craniofacial growth, and is strongly associated with symptoms of sleep fragmentation [8]. Children who predominantly breathe orally may exhibit snoring, restless sleep, fatigue, diminished concentration, and irritability [9]. These manifestations overlap significantly with the clinical presentation of ADHD, creating challenges in distinguishing behavioral disorders secondary to sleep impairment from primary neurodevelopmental conditions. Despite extensive evidence on SDB in community pediatric samples, studies evaluating behavioral outcomes specifically within orthodontic cohorts remain limited [10].

Recent epidemiological studies indicate that 25–50% of children diagnosed with ADHD exhibit concomitant sleep disturbances, while up to half of children with SDB present ADHD-like symptoms [3]. This bidirectional overlap suggests shared underlying mechanisms, which may include intermittent hypoxia, disturbed sleep architecture, neural inflammation, and impaired executive functioning [11,12]. In addition, SDB has been associated with oppositional-defiant behaviors, anxiety, mood dysregulation, and impaired social functioning [13,14].

Despite the increasing recognition of SDB as a contributor to behavioral problems, screening is not routinely integrated in orthodontic or dental settings [15]. The Pediatric Sleep Questionnaire (PSQ), a validated parent-reported tool, provides a practical and accessible method for identifying children at elevated risk [16,17]. Its use in orthodontic cohorts may help clinicians identify patterns suggestive of airway compromise or behavioral dysregulation early in the diagnostic process [15]. Given the frequent coexistence of oral habits, craniofacial imbalances, and potential airway obstruction in orthodontic patients, evaluating the relationship between SDB and behavioral symptoms is of particular clinical importance [18]. Understanding these associations may support interdisciplinary management strategies and reduce the risk of misdiagnosis. This clinical overlap highlights the importance of interdisciplinary collaboration among orthodontists, pediatric dentists, sleep physicians, and behavioral health professionals [5]. Data from Romanian pediatric orthodontic populations remain limited, despite the clinical relevance of airway-related functional habits and craniofacial phenotypes in everyday orthodontic practice.

Therefore, the present cross-sectional study aimed to determine the prevalence of SDB in a cohort of pediatric orthodontic patients and to examine its associations with oral habits and behavioral symptoms, including ADHD subtypes, oppositional-defiant traits, and anxiety/depression.

## 2. Materials and Methods

### 2.1. Study Design and Participants

A multicenter cross-sectional observational study was conducted in orthodontic clinics in Oradea and Târgu Mureș, Romania, between February 2024 and June 2025. The study included 186 consecutive children aged 7–13 years whose parents completed all required questionnaires. All participants were recruited during routine orthodontic consultations at the two participating centers. Children outside the target age range or with incomplete questionnaire data were excluded from the final analysis. Because the final research database retained only fully analyzable cases, the exact number of initially screened but excluded participants with incomplete data was not available. Demographic information collected included age, sex, residence (urban/rural), family structure, and weight category.

### 2.2. Data Collection Instruments

Parents completed three standardized instruments:

(1) Oral habits and demographics questionnaire
  This instrument recorded the presence, type, and reported duration of common oral habits, including mouth breathing, thumb or finger sucking, pacifier use beyond age four, lip sucking, atypical or infantile swallowing, onychophagia, bruxism, mandibular postural habits, and other parafunctional behaviors.
(2) Pediatric Sleep Questionnaire (PSQ)
  The PSQ is a 22-item parent-reported questionnaire designed to screen for sleep-related breathing disorders in children. Items address snoring, breathing irregularities during sleep, daytime sleepiness, and related behavioral manifestations. SDB was defined as 8 or more positive responses out of 22 PSQ items. In its original validation, the PSQ/SRBD scale demonstrated sensitivity values of 0.81–0.85 and specificity of 0.87 for identifying pediatric sleep-related breathing disorders [16,17]. Snoring was recorded both through the PSQ and as a separate variable when reported by parents.
(3) Vanderbilt ADHD Diagnostic Parent Rating Scale (VADPRS)
  Behavioral symptoms were assessed using the parent version of the Vanderbilt ADHD Diagnostic Rating Scale (VADPRS), a DSM-based parent-report screening instrument with acceptable psychometric properties and established utility in identifying ADHD-related symptom domains and common behavioral comorbidities [19,20]. The scale includes symptom domains for inattentive ADHD, hyperactive/impulsive ADHD, oppositional-defiant disorder (ODD), conduct disorder, and anxiety/depression. For the purposes of this study, the parent-form symptom-count thresholds were used to define positive screening profiles: ≥6/9 inattentive items for ADHD-inattentive symptoms, ≥6/9 hyperactive/impulsive items for ADHD-hyperactive/impulsive symptoms, both thresholds for ADHD-combined symptoms, ≥4/8 items for ODD, ≥3/14 items for conduct disorder, and ≥3/7 items for anxiety/depression. Because the scale was used as a screening measure in a questionnaire-based observational study and relied solely on parental reports, these outcomes were interpreted as screening-defined symptom profiles rather than clinical psychiatric diagnoses.

### 2.3. Clinical Examination

Standardized intraoral and extraoral examinations were performed by trained pediatric dentists across both centers. Malocclusion was classified in sagittal, transverse, and vertical planes using established orthodontic criteria. These variables were recorded for cohort characterization but were not analyzed in the present report. All procedures were conducted as part of routine clinical care and did not involve any additional interventions beyond standard clinical practice. Prospective orthodontic studies have documented measurable changes in airway dimensions following orthodontic interventions, supporting the relevance of craniofacial phenotyping in airway research [21].

### 2.4. Statistical Analysis

Descriptive statistics were used to summarize demographic characteristics, oral habits, SDB prevalence, and behavioral outcomes. Continuous variables were summarized as means and standard deviations (SDs), where applicable, and categorical variables are presented as frequencies and percentages. Participants with incomplete questionnaire data were excluded before analysis; therefore, a complete-case approach was used and no imputation procedures were applied.

Weight status categories were derived from BMI-based classifications recorded in the clinical charts at the participating centers and were used descriptively and as adjustment covariates. Because the original database did not retain the specific external pediatric growth reference used at the time of recording, these categories were not interpreted as standardized WHO- or CDC-derived epidemiologic classifications. Associations between SDB (defined as 8 or more positive responses on the 22-item PSQ) and behavioral variables were assessed using the chi-square (χ^2^) test or Fisher’s exact test, as appropriate. Because sparse cells were present for some outcomes, exact tests were applied where necessary. A significance threshold of *p* < 0.05 was adopted, and all statistical tests were two-tailed. Weight status was entered in the adjusted models as an ordinal variable (underweight, normal weight, overweight, grade I obesity). For sparse contingency tables, including ADHD-inattentive symptoms and conduct disorder, Fisher’s exact test was used. As a sensitivity analysis for the primary univariate behavioral comparisons, a Benjamini–Hochberg false discovery rate correction was applied. Multicollinearity was assessed using variance inflation factors, and model fit for the adjusted models was evaluated using the Hosmer–Lemeshow goodness-of-fit test.

To evaluate the independent association between SDB and behavioral outcomes, multivariable logistic regression analyses were performed. Covariates were selected a priori based on clinical relevance and included age, sex, and weight status. Adjusted odds ratios (ORs) with 95% confidence intervals (CIs) were calculated. Multivariable models were restricted to outcomes with analyzable event counts. Interaction terms were not tested because of the limited number of SDB-positive participants and the sparse-event structure of some behavioral outcomes. All analyses were conducted using IBM SPSS Statistics version 22 (IBM Corp., Armonk, NY, USA).

### 2.5. Ethics

The study was conducted in accordance with the Declaration of Helsinki. Written informed consent was obtained from the parents or legal guardians of all participating children, and all questionnaires were completed anonymously. At the participating orthodontic centers, non-interventional questionnaire-based studies using anonymized data collected as part of routine clinical care were not subject to formal ethics committee review under the institutional procedures governing observational clinical documentation at the time of data collection.

## 3. Results

### 3.1. Participant Characteristics

**Figure 1:**
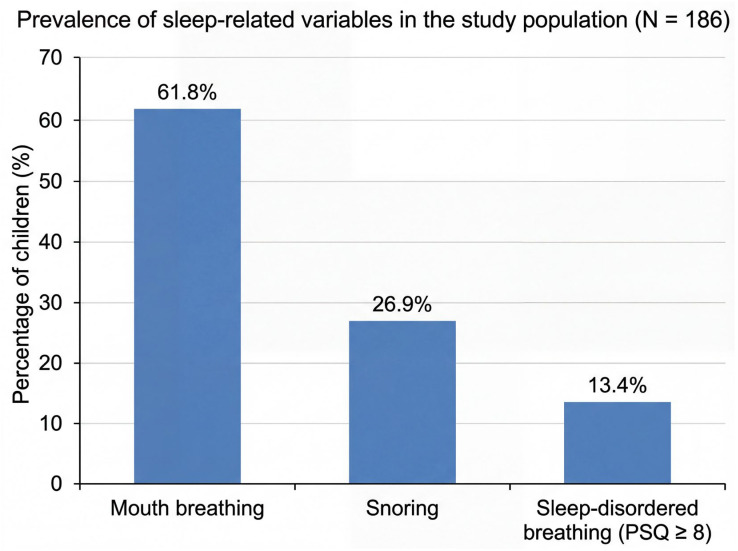
Prevalence of key sleep-related variables among pediatric orthodontic patients (N = 186). Bars represent the percentage of participants with mouth breathing (N = 115), snoring (N = 50), and PSQ-defined sleep-disordered breathing (8 or more positive responses out of 22 PSQ items; N = 25).

A total of 186 children aged 7–13 years were included in the analysis. The final sample was equally distributed across the two participating centers (93 children from each site). The cohort was slightly female-predominant and consisted mainly of urban participants from biparental families. Most children had normal weight status. Sleep-related symptoms were common, with mouth breathing being the most prevalent finding, followed by snoring and PSQ-defined sleep-disordered breathing. Detailed participant characteristics are presented in Table 1, and the prevalence of key sleep-related variables is illustrated in Figure 1.

### 3.2. Behavioral Outcomes

**Figure 2:**
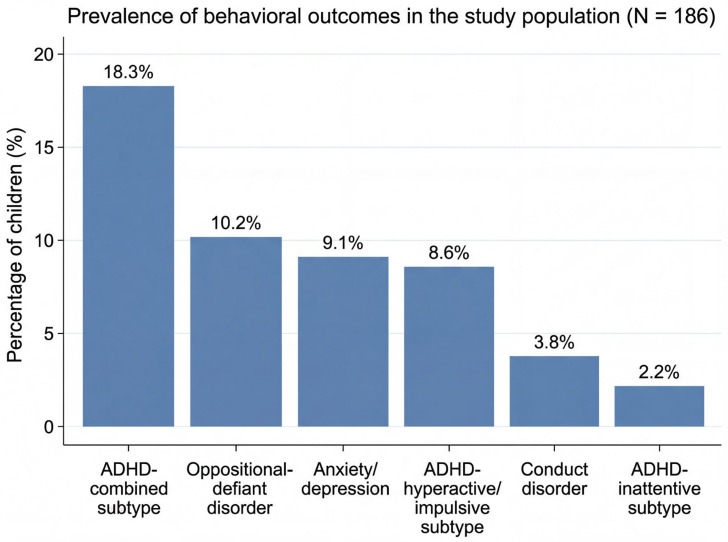
Prevalence of screening-defined behavioral symptom profiles among pediatric orthodontic patients (N = 186), based on parent-reported Vanderbilt ADHD Diagnostic Rating Scale measures. Bars represent the percentage of participants with positive screening profiles for ADHD-combined symptoms (N = 34), oppositional-defiant disorder symptoms (N = 19), anxiety/depression symptoms (N = 17), ADHD-hyperactive/impulsive symptoms (N = 16), conduct disorder symptoms (N = 7), and ADHD-inattentive symptoms (N = 4).

Behavioral screening identified a predominance of ADHD-related symptom patterns, with the combined subtype being the most frequent (18.3%, N = 34), followed by oppositional-defiant disorder (10.2%, N = 19) and anxiety/depression (9.1%, N = 17). The distribution of behavioral outcomes is illustrated in Figure 2, and detailed frequencies are presented in Table 2.

### 3.3. Associations Between Sleep-Disordered Breathing and Behavioral Symptoms

**Figure 3:**
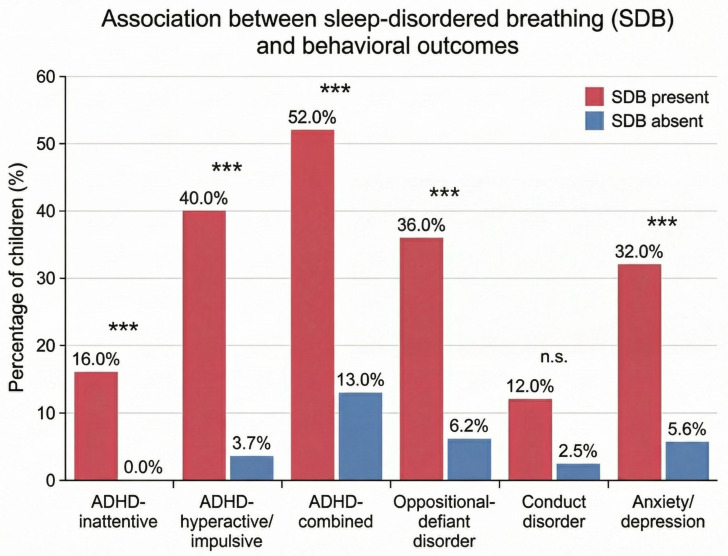
Frequency of screening-defined behavioral symptom profiles among children with PSQ-defined sleep-disordered breathing (SDB; N = 25) and those without SDB (N = 161). Bars represent percentages within each group. Positive screening profiles for ADHD-inattentive symptoms, ADHD-hyperactive/impulsive symptoms, ADHD-combined symptoms, oppositional-defiant disorder symptoms, and anxiety/depression symptoms were significantly more frequent in children with SDB. Conduct disorder showed a non-significant trend toward higher frequency in the SDB group. Statistical significance is indicated as follows: *** *p* < 0.001; n.s., not significant.

Children who screened positive for SDB (N = 25) demonstrated significantly higher frequencies of behavioral symptom profiles compared with those without SDB (N = 161) (Table 3). These differences are visually illustrated in Figure 3, highlighting the marked increase in ADHD-related symptoms, oppositional-defiant disorder, and anxiety/depression among children with SDB. Statistically significant associations were observed between SDB and ADHD-inattentive symptoms (16.0% vs. 0.0%, Fisher’s exact *p* = 0.0003), ADHD-hyperactive/impulsive symptoms (40.0% vs. 3.7%, *p* < 0.001), ADHD-combined subtype (52.0% vs. 13.0%, *p* < 0.001), oppositional-defiant disorder (36.0% vs. 6.2%, *p* < 0.001), and anxiety/depression (32.0% vs. 5.6%, *p* < 0.001). Conduct disorder was more frequent in children with SDB than in those without SDB (12.0% vs. 2.5%), but this difference did not reach statistical significance (Fisher’s exact *p* = 0.052). In a sensitivity analysis, adjustment for multiple comparisons using the Benjamini–Hochberg procedure did not materially alter the overall pattern of significant findings.

### 3.4. Multivariable Analysis

Multivariable logistic regression models were fitted to evaluate the association between sleep-disordered breathing (SDB) and behavioral outcomes, adjusting for age, sex, and weight status. SDB remained independently associated with ADHD-hyperactive/impulsive symptoms (OR = 5.84, 95% CI: 1.58–21.55, *p* = 0.008), ADHD-combined subtype (OR = 6.22, 95% CI: 2.19–17.65, *p* < 0.001), oppositional-defiant disorder (OR = 4.91, 95% CI: 1.44–16.76, *p* = 0.011), and anxiety/depression (OR = 4.38, 95% CI: 1.23–15.54, *p* = 0.022). Because of sparse-event structures, ADHD-inattentive symptoms could not be reliably modeled due to zero events in the non-SDB group, and conduct disorder was not included in the final adjusted models because of the low number of events. Adjusted regression results for analyzable outcomes are presented in Table 4.

## 4. Discussion

This cross-sectional study in a pediatric orthodontic cohort found that 13.4% of children screened positive for sleep-disordered breathing (SDB) based on the Pediatric Sleep Questionnaire threshold, while mouth breathing (61.8%) and snoring (26.9%) were frequently reported. Children who screened positive for SDB exhibited markedly higher frequencies of ADHD-related symptoms across all subtypes, oppositional-defiant behavior, and anxiety/depression compared with children who did not screen positive (Table 3). Overall, these findings are consistent with contemporary literature indicating that pediatric SDB is strongly associated with neurobehavioral morbidity and that sleep-related respiratory symptoms may contribute to behavioral phenotypes overlapping with ADHD and emotional dysregulation, thereby complicating recognition when sleep is not routinely assessed [1,2].

### 4.1. Interpretation of Findings

In our cohort, behavioral symptom frequencies were consistently higher among children with SDB than among those without SDB (Table 3), including marked between-group differences for hyperactive/impulsive ADHD, combined ADHD, oppositional-defiant behavior, and anxiety/depression. This pattern is in agreement with the well-established clinical overlap between sleep-related breathing disturbance and daytime behavioral dysregulation described in pediatric sleep medicine [3,10]. These differences are visually illustrated in Figure 3, highlighting the magnitude of behavioral disparities between children with and without SDB.

The 13.4% positive screening prevalence observed in the present study lies within the range reported in questionnaire-based orthodontic cohorts and is broadly compatible with recent evidence syntheses focused on children and adolescents referred for orthodontic assessment [22,23]. A recent systematic review in orthodontic settings emphasized that positive SDB screening prevalence varies substantially according to the instrument used, clinical phenotype, and study population, but remains sufficiently frequent to justify systematic screening in orthodontic care [22].

The very high prevalence of mouth breathing in our sample likely reflects both the general burden of oral deleterious habits in childhood and the “enriched” nature of orthodontic populations, in whom craniofacial disharmonies and functional habits are common reasons for referral [23,24]. Recent meta-analytic evidence suggests that oral deleterious habits remain highly prevalent in children worldwide, with mouth breathing among the most frequently reported behaviors [24]. This context is relevant because persistent mouth breathing is not merely a habit but may also represent a clinically visible marker of altered airway function.

Importantly, mouth breathing and related functional patterns, including atypical swallowing, are consistently linked to dentofacial development and malocclusion, and may interact bidirectionally with airway patency when they persist beyond early childhood [7,8]. Within interdisciplinary pediatric obstructive sleep apnea frameworks, orthodontic clinicians are increasingly recognized as key observers of phenotypes such as transverse maxillary constriction, altered mandibular posture, and craniofacial patterns associated with upper-airway vulnerability and sleep fragmentation [5,13].

### 4.2. Comparison with Previous Research

Population-based and guideline-oriented literature describe pediatric SDB as a spectrum ranging from primary snoring to obstructive sleep apnea, with prevalence estimates varying according to age, methodology, and diagnostic definition [25]. Although questionnaire-based screening cannot replace objective sleep assessment, it remains clinically valuable for identifying children at higher risk who may otherwise remain unrecognized [16,17].

The magnitude and consistency of associations between SDB and behavioral symptoms in our cohort are in line with systematic reviews and meta-analyses showing that children and adolescents with SDB experience more neurobehavioral deficits, including attention problems, hyperactivity, and broader behavioral impairment [1,2]. Contemporary reviews focused specifically on ADHD have likewise emphasized that SDB can produce ADHD-like daytime manifestations and that sleep-related contributors should always be considered when behavioral complaints are prominent [3,10].

Our findings also extend beyond ADHD-related symptoms by demonstrating significant associations between SDB and oppositional-defiant behavior as well as anxiety/depression. This is compatible with previous studies linking sleep-related breathing disturbance and sleep fragmentation to both externalizing and internalizing behavioral domains [2,9,13]. Longitudinal cohort data further suggest that parent-reported SDB symptoms in early childhood are associated with increased behavioral problems, supporting the view that sleep/airway dysfunction may influence emotional and behavioral development well before adolescence [13]. Likewise, clinical pediatric samples continue to report a higher behavioral burden among children with sleep-disordered breathing [9].

From a practical standpoint, the overlap between SDB symptomatology and behavioral syndromes remains a major challenge because it can increase diagnostic ambiguity and delay appropriate referral [3,10]. Observational pediatric care data have identified a strong association between ADHD medication use and positive screening for sleep-related breathing disorder “red flags,” highlighting how airway-related sleep problems may be overlooked when behavioral concerns dominate the clinical picture [26]. For orthodontic and dental clinicians, this reinforces the value of including simple sleep-related questions alongside routine craniofacial and oral-habit assessment. Because of the cross-sectional design, temporal ordering between sleep-disordered breathing and behavioral symptoms cannot be established, and reverse causation cannot be excluded.

Finally, the treatment literature strengthens the plausibility that improving airway obstruction and sleep quality can translate into better behavioral outcomes in at least a subset of children. Randomized trials of adenotonsillectomy in pediatric sleep apnea and mild sleep-disordered breathing have shown improvements in symptom burden and several patient-centered outcomes, including behavior and quality of life, even when some neurocognitive endpoints remain less responsive [27,28]. Together, these data support the interpretation that the associations observed in orthodontic cohorts are clinically meaningful and merit structured recognition and referral pathways [5].

### 4.3. Biological Mechanisms

Neurological pathways plausibly link SDB to behavioral dysregulation in children. Current pediatric sleep literature emphasizes the role of sleep fragmentation and intermittent hypoxia, which can disrupt restorative sleep architecture and alter neural systems involved in attention, executive functioning, impulse control, and emotional regulation [1,2]. These processes may help explain why the behavioral profile of children with SDB often resembles or overlaps with ADHD. Importantly, the main associations observed in the present study remained significant after adjustment for age, sex, and weight status, as shown in the multivariable analysis (Table 4).

Inflammatory and metabolic pathways have also emerged as candidate mediators of SDB-related neurobehavioral morbidity. Reviews focused on pediatric SDB increasingly suggest that vulnerability is heterogeneous and may depend not only on disease severity and duration, but also on biologic susceptibility, including inflammatory activation and oxidative imbalance [3,29]. This framework is relevant to the present study because the observed associations extended beyond attentional symptoms into oppositional-defiant behavior and anxiety/depression, suggesting that SDB-related daytime consequences may involve broader neurobehavioral systems. In addition, metabolic context may modulate hypoxia-related burden in sleep-related breathing disorders; in adults with obstructive sleep apnea, higher glycated hemoglobin has been associated with altered relationships among the apnea–hypopnea index and oxygenation indices, underscoring the complexity of hypoxia-linked physiological stress [30].

Within orthodontic settings, airway-relevant phenotypes may be amplified by persistent functional habits, particularly mouth breathing, and by dentofacial patterns that influence nasal resistance and upper-airway mechanics [5,7,8,31]. Systematic reviews and meta-analyses have shown that chronic mouth breathing is associated with altered facial skeletal development, atypical swallowing, and dentofacial morphology, providing a biologically plausible pathway linking chronic oral breathing to both craniofacial patterning and sleep-related airway vulnerability [7,8]. Three-dimensional imaging studies have also demonstrated oropharyngeal airway changes after orthopedic interventions, underscoring the potential for orthodontic treatment to modify airway mechanics and the importance of integrating imaging phenotypes in future prospective work [32].

### 4.4. Adjunctive Approaches and Translational Considerations

Current guidance emphasizes that pediatric SDB management should remain interdisciplinary and airway-focused, with referral for definitive evaluation when clinically indicated [25]. In many cases, adenotonsillectomy remains a key option when adenotonsillar hypertrophy is contributory, and randomized studies support improvement in symptom burden and patient-centered outcomes following airway-focused treatment [27,28]. Orthodontic and dentofacial interventions may also be relevant in selected phenotypes, particularly where craniofacial structure contributes to airway compromise [5,33,34].

Beyond structural interventions, there is growing interest in adjunctive strategies targeting stress-related, inflammatory, and oxidative pathways that may influence symptom burden and downstream neurobehavioral sequelae. In dental and oral-surgical settings, lavender-based interventions have been explored for anxiety and peri-procedural symptom control, although findings remain heterogeneous and derive from contexts distinct from pediatric SDB [35,36,37,38,39,40]. At a broader level, systematic reviews and experimental studies support modest anxiolytic, anti-inflammatory, and antioxidant properties of lavender-based interventions and *Lavandula angustifolia* derivatives [41,42,43,44,45,46]. However, these data do not directly address pediatric sleep-disordered breathing and were not evaluated in the present cohort; they should therefore be regarded as hypothesis-generating only, and any future role in sleep-related behavioral disturbances remains to be established.

### 4.5. Strengths and Limitations

Strengths of this study include the multicenter design, consecutive recruitment within routine orthodontic practice, the use of validated screening instruments, and the consistency of associations across multiple behavioral domains. The Pediatric Sleep Questionnaire remains a practical and validated parent-reported instrument for identifying children at increased risk of sleep-related breathing disorder in both clinical and research settings [16,17].

However, several limitations should be acknowledged. Although it is balanced across two centers, the cohort represents an enriched orthodontic clinical sample rather than a community-based pediatric population, which may limit external validity and does not fully exclude center-related differences in case mix. The cross-sectional design precludes causal inference and does not allow temporal ordering between sleep-related breathing symptoms and behavioral symptom profiles to be established. All sleep-related and behavioral data were parent-reported, introducing the possibility of reporting bias, and behavioral outcomes were based on screening instruments rather than clinical psychiatric diagnoses. In addition, socioeconomic status and parental education were not available in the study dataset and therefore could not be examined as potential confounders. Because data were collected during routine orthodontic consultations, parental concern regarding oral habits, breathing patterns, or behavioral symptoms at the time of assessment may also have influenced questionnaire responses. Moreover, PSQ-based screening does not replace polysomnographic diagnosis, and the relatively small number of SDB-positive participants limits statistical power for less frequent outcomes and may affect the stability of multivariable estimates. Despite these limitations, the consistency and magnitude of the observed associations suggest that orthodontic settings may represent a meaningful opportunity for earlier recognition of children with overlapping airway and behavioral concerns.

### 4.6. Future Directions

Future studies should combine orthodontic phenotyping with objective sleep assessment, including polysomnography or other validated sleep measures, in order to clarify the temporal and dose–response relationships between airway dysfunction and behavioral outcomes [25,29]. Multicenter prospective studies would also help determine which craniofacial and functional phenotypes carry the greatest neurobehavioral risk and which treatment pathways are most likely to produce clinically meaningful improvements. Integrating imaging-based airway phenotypes may be particularly valuable in this context [32].

Implementation research is likewise needed to define feasible screening pathways within orthodontic practice, integrating validated questionnaires with clinical examination and referral algorithms so that children at high risk can be identified earlier and directed to appropriate multidisciplinary care [5,22]. Finally, future translational work may investigate inflammatory, oxidative stress, and metabolic biomarkers, together with emerging assessment strategies that combine biomarkers, biosignals, and intelligent screening approaches, to better explain inter-individual variability in neurobehavioral vulnerability and treatment response [29,30,47].

## 5. Conclusions

This study supports the clinical relevance of sleep-disordered breathing screening in pediatric orthodontic patients. In this multicenter cohort, SDB was identified in a meaningful proportion of children and was significantly associated with multiple screening-defined behavioral symptom domains, particularly ADHD-related symptoms, oppositional-defiant behavior, and anxiety/depression. Mouth breathing and snoring were also common findings, reinforcing the orthodontic setting as a clinically valuable point for early recognition of airway-related dysfunction.

These findings suggest that behavioral symptoms observed in orthodontic patients should not be interpreted in isolation, particularly when accompanied by oral breathing patterns or sleep-related complaints. Incorporating brief airway- and sleep-oriented screening tools, such as the Pediatric Sleep Questionnaire, into routine orthodontic assessment may facilitate earlier identification of children at risk and support timely interdisciplinary referral. Given the cross-sectional and questionnaire-based nature of the study, the results should be interpreted as associative rather than causal. Prospective studies integrating objective sleep assessment and craniofacial phenotyping are warranted to clarify the biological and clinical pathways linking airway dysfunction and neurobehavioral outcomes in children.

## Figures and Tables

**Table 1:** Participant characteristics.

Variable	Category	N (%)
Age (years)	Mean ± SD	9.99 ± 1.99
Sex	Female	102 (54.8)
	Male	84 (45.2)
Residence	Urban	161 (86.6)
	Rural	25 (13.4)
Family structure	Biparental	158 (84.9)
	Single-parent	28 (15.1)
Weight status	Underweight	16 (8.6)
	Normal weight	124 (66.7)
	Overweight	40 (21.5)
	Grade I obesity	6 (3.2)
Snoring	Yes	50 (26.9)
Mouth breathing	Yes	115 (61.8)
Sleep-disordered breathing (SDB)	PSQ ≥ 8	25 (13.4)

**Table 2:** Behavioral outcomes in the study population.

Behavioral Outcome	N (%)
ADHD-inattentive subtype	4 (2.2)
ADHD-hyperactive/impulsive subtype	16 (8.6)
ADHD-combined subtype	34 (18.3)
Oppositional-defiant disorder (ODD)	19 (10.2)
Conduct disorder	7 (3.8)
Anxiety/depression	17 (9.1)

**Table 3:** Association between SDB and behavioral outcomes.

Behavioral Outcome	SDB Present (N = 25)	SDB Absent (N = 161)	*p*-Value
ADHD-inattentive	4 (16.0%)	0 (0.0%)	0.0003
ADHD-hyperactive/impulsive	10 (40.0%)	6 (3.7%)	<0.001
ADHD-combined	13 (52.0%)	21 (13.0%)	<0.001
Oppositional-defiant disorder	9 (36.0%)	10 (6.2%)	<0.001
Conduct disorder	3 (12.0%)	4 (2.5%)	0.052
Anxiety/depression	8 (32.0%)	9 (5.6%)	<0.001

*p*-values were obtained using chi-square or Fisher’s exact test, as appropriate. Fisher’s exact test was applied for sparse-cell outcomes, including ADHD-inattentive symptoms and conduct disorder.

**Table 4:** Multivariable logistic regression analysis of the association between SDB and behavioral outcomes.

Outcome	Adjusted OR	95% CI	*p*-Value
ADHD-hyperactive/impulsive	5.84	1.58–21.55	0.008
ADHD-combined	6.22	2.19–17.65	<0.001
Oppositional-defiant disorder	4.91	1.44–16.76	0.011
Anxiety/depression	4.38	1.23–15.54	0.022

Adjusted odds ratios (ORs) were derived from multivariable logistic regression models including age, sex, and weight status. Models were fitted only for outcomes with sufficient analyzable event counts. ADHD-inattentive symptoms could not be reliably modeled because of zero events in the non-SDB group, and conduct disorder was not included in the final adjusted models because of sparse events.

## Data Availability

The data presented in this study are available on request from the corresponding author due to privacy, ethical, and institutional data-protection restrictions related to pediatric participants. Requests for access will be considered for scientifically justified purposes and subject to appropriate de-identification safeguards. A data dictionary can be provided upon reasonable request.

## Abbreviations

The following abbreviations are used in this manuscript:

ADHD	Attention-Deficit/Hyperactivity Disorder
ODD	Oppositional-Defiant Disorder
PSQ	Pediatric Sleep Questionnaire
SDB	Sleep-Disordered Breathing
OSA	Obstructive Sleep Apnea
SRBD	Sleep-Related Breathing Disorder
